# Leveraging epileptic network understanding to improve targeted treatment

**DOI:** 10.1371/journal.pbio.3003625

**Published:** 2026-02-13

**Authors:** James E. Niemeyer

**Affiliations:** Department of Neurosurgery, Weill Cornell Medicine, New York, New York, United States of America

## Abstract

Epilepsy is thought to develop through pathological connections involving various and widespread brain regions. This primer discusses a PLOS Biology study in mice that shows how some of these connections are formed across network sites, and how this interconnectivity can serve as a treatment target in epilepsy.

## Teaser

Epilepsy is thought to develop through pathological connections involving various and widespread brain regions. A new study in *PLOS Biology* shows how some of these connections are formed across network sites, and how this interconnectivity can serve as a treatment target in epilepsy.

Roughly 1 in 3 patients with epilepsy will exhibit drug resistance [1]. While surgical resection and neurostimulator treatments can be effective in these cases, many patients continue to experience some recurring seizures [2]. These treatments typically target seizure onset zones or deep relay sites, but researchers have increasingly probed the possibility of interventions that consider more specific epileptic network regions, including manipulation of sites connected to, but outside of, the seizure onset zone [3,4]. However, identifying the most targetable and vulnerable regions of these “seizure networks” is made difficult by the exceptionally broad and complex connectivity of the brain [2,5]. In a new study published in *PLOS Biology*, Tao and colleagues [6] perform a wide array of experiments to dissect a particular network involved in seizure spread, identifying how cell-type-specific connectivity may underly the “functional connectivity”—the coordinated patterns of neural activity across the brain—in epilepsy, as well as how these connections could be targeted for future treatments.

Tao and colleagues performed their work in a rodent epilepsy model with seizures initiating in the anterior piriform cortex of mice, a region of the mammalian brain that contains an infamous site referred to as the area tempestas (Latin for “stormy area”) [7,8]. Piriform cortex is well-connected with various brain regions, positioning it as an ideal hub to broadcast pathological activity efficiently and quickly through the brain. However, the specific connections of this site, the cell types involved, and their impact on functional connectivity in the brain during epilepsy development are not fully understood. To address this, Tao and colleagues applied repeated optogenetic stimulation of excitatory cells in the piriform cortex of mice and then performed fMRI, electrophysiology, calcium imaging, and circuit manipulations to determine how these cells inform epilepsy development and how certain connections may be targeted to prevent seizures.

The authors first used repeated optogenetic stimulation to “kindle” the piriform cortex of mice over several days, ultimately producing generalized seizures. By restricting stimulation to excitatory projection neurons, they sought to delineate the downstream regions most impacted by pathological activity developing in the piriform cortex. The authors then applied fMRI and found functional connectivity increases in various widespread brain regions, with a significant and dominant increase in connectivity with the lateral entorhinal cortex. Viral tracing confirmed that this anatomical pathway consisted primarily of piriform excitatory cells projecting to lateral entorhinal cortex excitatory cells. To test the significance of this connection in seizure propagation, the authors next blocked neurotransmission between the piriform and lateral entorhinal cortices. This resulted in near elimination of epileptiform activity and generalized seizures, as well as significantly weakened calcium signals, following piriform stimulation. Meanwhile, inhibition of other pathways, involving the perirhinal cortex or basolateral amygdala, did not prevent generalized seizures, though some partial suppression was noted. Importantly, inhibition of regions with clear increased functional connectivity but lacking direct anatomical projections to piriform cortex, such as motor and retrosplenial cortices, did not block generalized seizures. These experiments demonstrated that specifically inhibiting excitatory projections from a seizure onset hub can prevent generalized seizures in this model. This finding by itself is not terribly surprising: the seizure onset zone has long been targeted in patients with epilepsy. However, the authors next asked whether subsequent connections of the network might also serve as treatment targets.

To test this, the researchers extended their work to a downstream connection of the network involving lateral entorhinal cortex projections to the dentate gyrus. This “second stage” of the epileptic network exhibited significant changes in resting state functional connectivity (again determined by fMRI) after kindling. By employing viral tracing, the researchers also noted dense fiber innervation of the molecular layer of the dentate gyrus. This was reaffirmed by calcium imaging studies showing that seizures in the piriform cortex would proceed sequentially through the lateral entorhinal cortex and then the dentate gyrus, with each of these network nodes being recruited about 2–4 s apart. Interestingly, the duration of the calcium responses was notably increased over multiple days of kindling across these three network sites, highlighting that generalized seizures within a network likely require progressive changes of connectivity between seizure network sites.

These data altogether suggested that connections between the lateral entorhinal cortex and dentate gyrus may also be targetable to impede network propagation of seizures emanating from the piriform cortex. To test this, the researchers again used viral methods to prevent neurotransmission specifically from lateral entorhinal cortical excitatory cells that projected to the dentate gyrus. Following disruption of this network connection, the authors observed significant decreases in seizure rates.

Altogether, this study demonstrates that inhibiting direct connections from a seizure onset zone in piriform cortex to the lateral entorhinal cortex can prevent generalized seizures, and that even inhibition of connections at *downstream* nodes (entorhinal cortex to dentate gyrus) can impede seizure propagation. These findings highlight how knowledge about epileptic networks, such as the specific connections and brain regions involved, may provide new spatial targets capable of preventing seizures. In human patients with epilepsy, these cell-type specific connection treatments are not yet available, though researchers are increasingly testing patient-specific targeting [4,9] and exploring how different forms of neurostimulation may preferentially recruit different cell types depending on parameters like stimulation frequency and waveform [10]. Thus, it is likely that future electrical stimulation treatments will increasingly consider broader networks, such as downstream connections, in treating patients with epilepsy.

**Fig 1:**
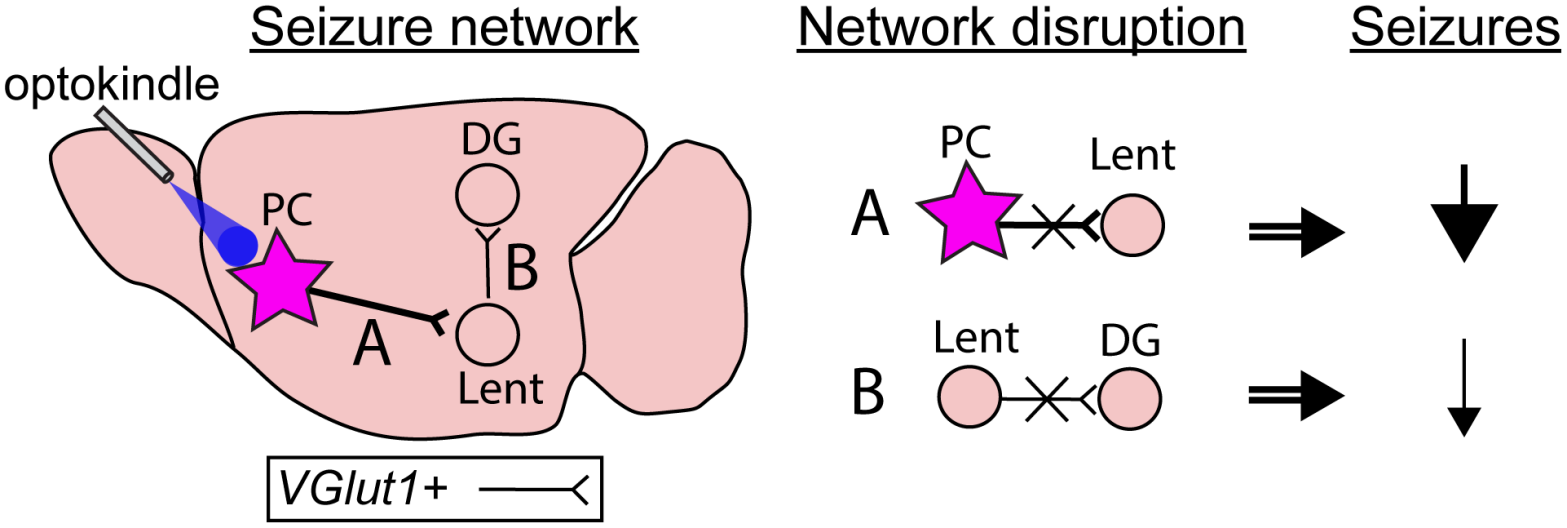
The anterior piriform cortex epileptic network model and targeting. The authors applied multi-day optogenetic kindling in mice to induce robust generalized seizures that initiated in the piriform cortex (PC). Over days, resting state functional connectivity changed across the brain, with a pronounced increase to lateral entorhinal cortex (Lent), the strongest direct anatomical projection target of the piriform cortex. The authors then disrupted the excitatory (*Vglut1+)* connections from PC to Lent, finding that this effectively prevented propagating seizures and induced brain-wide changes in functional connectivity. The Lent also exhibited excitatory projections to the dentate gyrus (DG). When this second-step of the network pathway was targeted, seizures decreased, but not as effectively as when the PC-Lent pathway was disrupted. This optokindling model presents a novel method of probing multi-day epileptic network formation coupled with on-demand seizures to test network-oriented treatments.

While the optogenetic stimulation model may have limited relevance to human epilepsy, this model shows that seizure network “recruitment” can occur progressively during epilepsy development. Though we expect that epileptic networks form over long time periods [2], the optogenetic kindling model here, using only several days, presents an intriguing opportunity to probe how specific connections could be disrupted during epilepsy development. For example, subsequent studies could apply the same network interventions to the lateral entorhinal cortex or dentate gyrus but at day 2 rather than day 4, perhaps even testing multi-site manipulations. Such follow-up work would be broadly relevant to the epilepsy research community as new stimulation methods, including noninvasive ones, are increasingly translated to humans. These future experiments, targeting the endogenous connectivity that is exploited by seizure networks, could be highly useful toward improving treatments in patients with drug-resistant epilepsy (Fig 1).

## Abbreviations

DG: dentate gyrus
Lent: lateral entorhinal cortex
PC: piriform cortex
